# Corps étranger métallique géant intraoculaire

**DOI:** 10.11604/pamj.2015.20.357.4333

**Published:** 2015-04-14

**Authors:** Rajae Derrar, Rajae Daoudi

**Affiliations:** 1 Université Mohammed V Souissi, Service d'Ophtalmologie A Hôpital des Spécialités CHU, Rabat, Maroc

**Keywords:** Corps étranger, baisse d′acuité, traumatisme, foreign body, decline acuity, trauma

## Image en medicine

**Figure 1:**
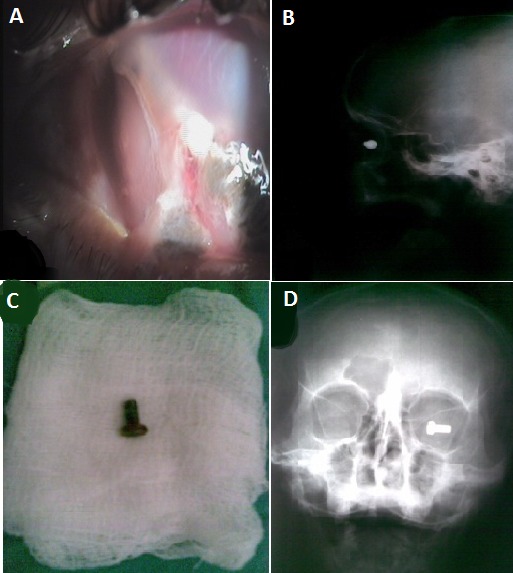
Corps étranger métallique intraoculaire causant une plaie de cornée avec hernie irienne

Patient âgé de 28 ans admis aux urgences ophtalmologiques pour baisse d'acuité majeure avec douleur oculaire suite à un traumatisme par projection oculaire d'un corps étranger. A l'examen, l'acuité visuelle est à perception lumineuse douteuse avec plaie de cornée, un segment antérieur remanié avec hernie de l'iris (A), l'examen du segment postérieur est impossible. Une radiographie standard des orbites de face et de profil ont été demandées et ont objectivé un corps étranger radio opaque ayant la forme d'une vis au niveau de l'orbite droite (B et D). Le patient a bénéficié d'une extraction à l’éléctroaimant du corps étranger géant intraoculaire qui s'est révélé être une vis (C) avec suture de la plaie cornéenne. L'acuité visuelle du patient s'est limitée à une perception lumineuse positive. Figure 1

